# All-age hospitalization rates in coal seam gas areas in Queensland, Australia, 1995–2011

**DOI:** 10.1186/s12889-016-2787-5

**Published:** 2016-02-06

**Authors:** Angela K. Werner, Kerrianne Watt, Cate M. Cameron, Sue Vink, Andrew Page, Paul Jagals

**Affiliations:** 1 Sustainable Minerals Institute, The University of Queensland, Sir James Foots Bldg (47a), Level 6, CWiMI, Corner Staffhouse and College Roads, St. Lucia, QLD 4072 Australia; 2 College of Public Health, Medical and Veterinary Sciences, James Cook University, Townsville, QLD Australia; 3 School of Public Health, The University of Queensland, Herston, QLD Australia; 4 CONROD Injury Research Centre, Menzies Health Institute Queensland, Griffith University, Meadowbrook, QLD Australia; 5 Centre for Health Research, Western Sydney University, Penrith, NSW Australia

**Keywords:** Coal seam gas, Environmental health impact, Hospital admissions, Queensland, Unconventional natural gas

## Abstract

**Background:**

Unconventional natural gas development (UNGD) is expanding globally, with Australia expanding development in the form of coal seam gas (CSG). Residents and other interest groups have voiced concerns about the potential environmental and health impacts related to CSG. This paper compares objective health outcomes from three study areas in Queensland, Australia to examine potential environmentally-related health impacts.

**Methods:**

Three study areas were selected in an ecologic study design: a CSG area, a coal mining area, and a rural/agricultural area. Admitted patient data, as well as population data and additional factors, were obtained for each calendar year from 1995 through 2011 to calculate all-age hospitalization rates and age-standardized rates in each of these areas. The three areas were compared using negative binomial regression analyses (unadjusted and adjusted models) to examine increases over time of hospitalization rates grouped by primary diagnosis (19 ICD chapters), with rate ratios serving to compare the within-area regression slopes between the areas.

**Results:**

The CSG area did not have significant increases in all-cause hospitalization rates over time for all-ages compared to the coal and rural study areas in adjusted models (RR: 1.02, 95 % CI: 1.00–1.04 as compared to the coal mining area; RR: 1.01, 95 % CI: 0.99–1.04 as compared to the rural area). While the CSG area did not show significant increases in specific hospitalization rates compared to both the coal mining *and* rural areas for any ICD chapters in the adjusted models, the CSG area showed increases in hospitalization rates compared only to the rural area for neoplasms (RR: 1.09, 95 % CI: 1.02–1.16) and blood/immune diseases (RR: 1.14, 95 % CI: 1.02–1.27).

**Conclusions:**

This exploratory study of all-age hospitalization rates for three study areas in Queensland suggests that certain hospital admissions rates increased more quickly in the CSG study area than in other study areas, particularly the rural area, after adjusting for key sociodemographic factors. These findings are an important first step in identifying potential health impacts of CSG in the Australian context and serve to generate hypotheses for future studies.

**Electronic supplementary material:**

The online version of this article (doi:10.1186/s12889-016-2787-5) contains supplementary material, which is available to authorized users.

## Background

The expansion of the coal seam gas (CSG) industry in Australia has raised concerns about potential human health impacts in part because of a current lack of human health impact assessment information, as well as accessible baseline studies in Australia [1]. Furthermore, information on exposures to CSG-associated environmental hazards is minimal. There is a need for source-to-effect pathways to be fully mapped for relevant exposure media, including air and soil. In the broader unconventional natural gas development (UNGD) context, there is generally a lack of health research on the effects of UNGD [2].

Some health-related studies have been conducted in other locations, predominantly in the United States. These include cross-sectional studies [3, 4], ecological [5], qualitative [6, 7], retrospective cohort [8], ‘difference-in-differences’ design [9], as well as human health risk assessments [10–12] and Health Impact Assessment (HIA) [13]. Such studies have provided some evidence for adverse health outcomes potentially associated with UNGD, but have suggested the need for further research. These studies have predominantly examined cancer incidence [5], birth outcomes [8, 9, 14], cancer and non-cancer risks for air emissions [10], and a range of other areas of concern identified in an HIA (including air pollution, water and soil contamination, and community wellness) [13, 15].

Investigation of the association between exposures associated with CSG extraction and health outcomes are often limited by the relatively small population base exposed (as CSG wells are often established in less populated rural areas). This is combined with a focus on often rare health outcomes with long latencies (such as cancer incidence), or common outcomes and/or syndromes that could plausibly be attributed to other putative causes occurring contemporaneously to CSG development.

Adgate et al. [2] noted that more epidemiological studies are needed to determine what disease patterns may exist and how UNGD may affect these patterns. This may be helpful for companies to improve practices to reduce exposures, or to assist in formulating stricter regulations where necessary. A recent literature review concluded that the majority of studies published on UNGD and environmental health concentrated on shale gas, focused only on a few key areas of environmental health, such air and water, and generally lacked methodological rigor [16]. Many of the UNGD-related studies have focused on shale gas, which means that these outcomes do not necessarily translate to the CSG context due to the differences between both types of UNGD [17].

To the authors’ knowledge, no epidemiological studies have been conducted on the impacts of CSG on human health, in Australia or elsewhere. This, as well as the level of public concern, prompted this exploratory study. The objective of this analysis was to explore trends in hospitalization rates for three designated geographical study areas (CSG, coal mining, rural) in Queensland over the period 1995–2011. The three study areas were used to determine whether there were increases in hospitalization rates for health conditions, as measured by ICD-10-AM codes, in the CSG area compared to the coal mining and rural study areas.

## Methods

### Ethics approval

Ethics approval was obtained from the University of Queensland Behavioural & Social Sciences Ethical Review Committee (approval number 2012000582). Access to confidential data was obtained via the Public Health Act through Queensland Health (approval number RD004515).

### Setting and study area classification

**Fig. 1:**
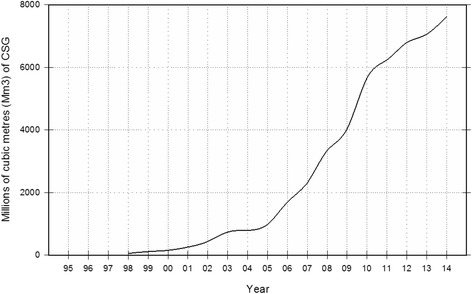
Queensland coal seam gas production (Mm^3^) over the study time period

Queensland is the second largest state in Australia, approximately 1.7 million km^2^ in size, and is home to 4.7 million people [18, 19]. The state has significant resource development, including coal mining and CSG extraction, with agricultural enterprises and tourism also adding to the predominant economic activities [20, 21]. In Queensland, the Bowen and Surat Basins are areas of major resource development, including coal mining and CSG development. Gas production from these basins represents over 88 % of the total gas produced in the state. Figure 1 shows coal seam gas production figures over the study period. Production started to increase in 2001/2002 and increasingly ramped up in 2005/2006.

The three study areas were selected to reflect three different areas of environmental impact activity and selection of these sites has been described elsewhere [22]. These areas were identified as a CSG area, a coal high-impact (CHI) area (where coal mining, but no CSG development was present), and a rural low-impact (RLI) area (where no coal mining or CSG development was present). All were non-metropolitan regions of Queensland (i.e., regional/rural). The three areas were grouped according to broad groupings of Statistical Local Areas (SLAs). SLAs are geospatial units reflecting local-area populations that cover Australia without any gaps or overlaps between areas [23], giving each area a unique SLA identifier.

The time period 1995–2011 was selected in order to include a period of time where all areas had minimal to no CSG activity, in particular, in the CSG area. The areas evolved over this time period, with the CSG study area increasing CSG development activities (Fig. 1). This study includes the cumulative effects over the entire time period.

Table 1 shows the demographic and socioeconomic characteristics for each study area during the study time period, including the proportion of the population who were Indigenous, as well as the proportion who were Australian-born, employed full-time, and other selected characteristics. As of 2011, the population in the CSG area was approximately 44 217 (average median age 39.0 years), 7747 in the RLI area (average median age 39.9 years), and 35 142 in the CHI area (average median age 30.2 years) [24].

**Table 1:** Demographic and socioeconomic characteristics used as model covariates for the coal seam gas, rural low-impact, and coal high-impact study areas for the start, mid-point, and end of the study time period (1995–2011)^a^

	CSG			RLI			CHI		
	1995	2003	2011	1995	2003	2011	1995	2003	2011
Population	40100	40529	44217	8804	8306	7747	32508	30644	35142
Percentage male	51.45	51.50	51.76	52.77	51.70	51.00	53.83	53.82	53.97
Percentage female	48.55	48.50	48.24	47.23	48.30	49.00	46.17	46.18	46.03
Percentage Indigenous persons	2.83	4.22	4.86	10.99	13.14	15.64	5.24	4.84	5.71
Percentage persons Australian-born	89.35	86.74	83.40	92.77	87.21	83.36	89.77	86.11	77.78
Percentage persons employed full-time	31.39	31.09	31.86	33.79	33.27	33.12	36.23	37.04	36.24
Percentage persons in managerial, administrative, or professional occupations	14.90	15.61	15.30	13.72	14.79	16.28	9.84	11.47	12.05
Weighted average median weekly household income	485.81	749.24	1135.58	475.15	696.97	946.35	926.27	1250.16	2182.04
Weighted average mean household size	2.71	2.56	2.50	2.71	2.45	2.31	3.14	3.0	2.99

^a^ *CSG* coal seam gas, *RLI* rural low-impact, *CHI* coal high-impact

### Data

Hospitalization data (defined as admitted to hospital for a period of 24 h or longer) were obtained from Queensland Health through the Queensland Hospital Admitted Patient Data Collection (QHAPDC). Data on age, date of hospital admission, and primary diagnosis code were obtained. Data were obtained for each calendar year for the period 1995–2011 for the three selected areas for admission to any hospital in Queensland for any resident of one of the three study areas. Thus, hospital admissions data were not obtained on people who were hospitalized in one of the three areas, but who were *not* a resident in one of those areas (e.g., fly-in, fly-out workers or tourists).

Two versions of International Classification of Diseases (ICD) coding were used for primary diagnosis codes during the study period: ICD-9-Australian Modification (ICD-9-AM), which was used for cases from 1995 to July 1999, and ICD-10-Australian Modification (ICD-10-AM), which was used from July 1999 through the remainder of the study period. Hence, ICD-9 codes were forward - mapped to equivalent ICD-10 codes for analyses. Hospitalization data in Queensland are episode-based, not patient-based, meaning each record was based on an episode of care for each formal separation. Therefore, each hospitalization episode may not represent unique individuals.

While there are 22 ICD chapters, only 19 ICD chapters were examined as primary diagnosis codes were of interest. Chapter 20 (*‘External causes’*) is often used in conjunction with *‘Injuries’*-related diagnoses and is used as supplementary information. Chapter 21 (*‘Factors influencing health status’*) is used for admissions not related to a disease or injury, and Chapter 22 (*‘Codes for special purposes’*) is used for provisional codes. Hence, the exclusion of these chapters due to the lack of primary diagnosis codes of interest (related to a disease or injury admission). The remaining 19 ICD chapters (primary diagnosis codes classified by ICD chapter) were included rather than selecting specific ICD chapters or codes a priori due to the dearth of information related to CSG-specific health impacts. Each ICD chapter that was examined included all of the relevant codes within a given chapter.

Population data were obtained from the Australian Bureau of Statistics (ABS). Estimated resident population (ERP) counts by sex and by age group (0–4 years, 5–9 years, 10–14 years, 15–19 years, 20–24 years, 25–34 years, 35–44 years, 45–54 years, 55–64 years, 65–74 years, 75–84 years, 85+ years) and study area were obtained for each year from 1995 to 2011, matched to the study areas for the hospital data. Age groups were grouped more broadly for the adjusted models to ensure sufficient numbers in each study area (i.e., 0–19 years, 20–64 years, and 65–85+ years). Population data were obtained on place of usual residence rather than place of enumeration, which allows the effects of factors, such as temporary living arrangements and travel, to be removed [25].

Covariate data were obtained from the ABS for each Census year (1991, 1996, 2001, 2006, and 2011) to account for potential socio-demographic differences between the study areas [24]. Data were collected for each SLA within the three study areas and aggregated to the identified CSG, CHI, or RLI areas, with intercensal years estimated using weighted interpolation. Covariates included: number of Indigenous persons, number of persons Australian-born, number of persons employed full-time, number of persons in white collar occupations (i.e., managerial and administrative, professionals), median household income, and mean household size. Weighted averages were obtained for household income and household size, while the remaining variables were calculated as the proportion of the population in each study area.

### Analysis

All-age hospitalization rates per 1000 persons were calculated for each calendar year for the period 1995–2011 for each study area. Crude all-cause rates were calculated for each study area. Secular trends in hospitalization rates were examined in the CSG, CHI, and RLI areas to identify ICD chapters where the CSG area showed increasing rates over time *and* where the patterns found in the CSG area differed from the CHI and RLI areas. Visual inspection allowed for understanding general trends, identifying outliers, as well as a preliminary assessment of similarities and differences across ICD chapters [26]. Direct age-standardized rates were calculated for all-cause admissions, and for 19 of the ICD chapters, using the 2001 Australian population as the standard population [27].

Counts were modeled in a series of negative binomial regression models, offset by the log of the population. The focus of this study was to assess potential health impacts of CSG via hospitalization rates. Therefore, of primary interest was whether hospitalization rates increased for any health condition (assessed via diagnoses within ICD chapter codes) over time in CSG areas relative to the changes in either, or both, of the other two study areas. Accordingly, time was included as a continuous ‘period’ variable, and the area and period interaction was used as an assessment of the relative change in slopes over time between areas.

Rate ratios (RR; 95 % CI) from these models were calculated to describe any relative increases over time in hospitalization rates for a particular health condition in the CSG area relative to the CHI area and to the RLI areas. Goodness of fit criteria (deviance, dispersion, AIC, and BIC) were examined to assess how well the models fit the data [28]. Base models were estimated (unadjusted), followed by models adjusting for age and sex, then models adjusting for additional identified covariates, including the proportion of Indigenous persons, proportion Australian-born, proportion employed full-time, proportion in white collar occupations (i.e., managerial and administrative, professionals), median household income, and mean household size. Regression modeling was carried out in PROC GENMOD using SAS 9.4 [29].

## Results

**Fig. 2:**
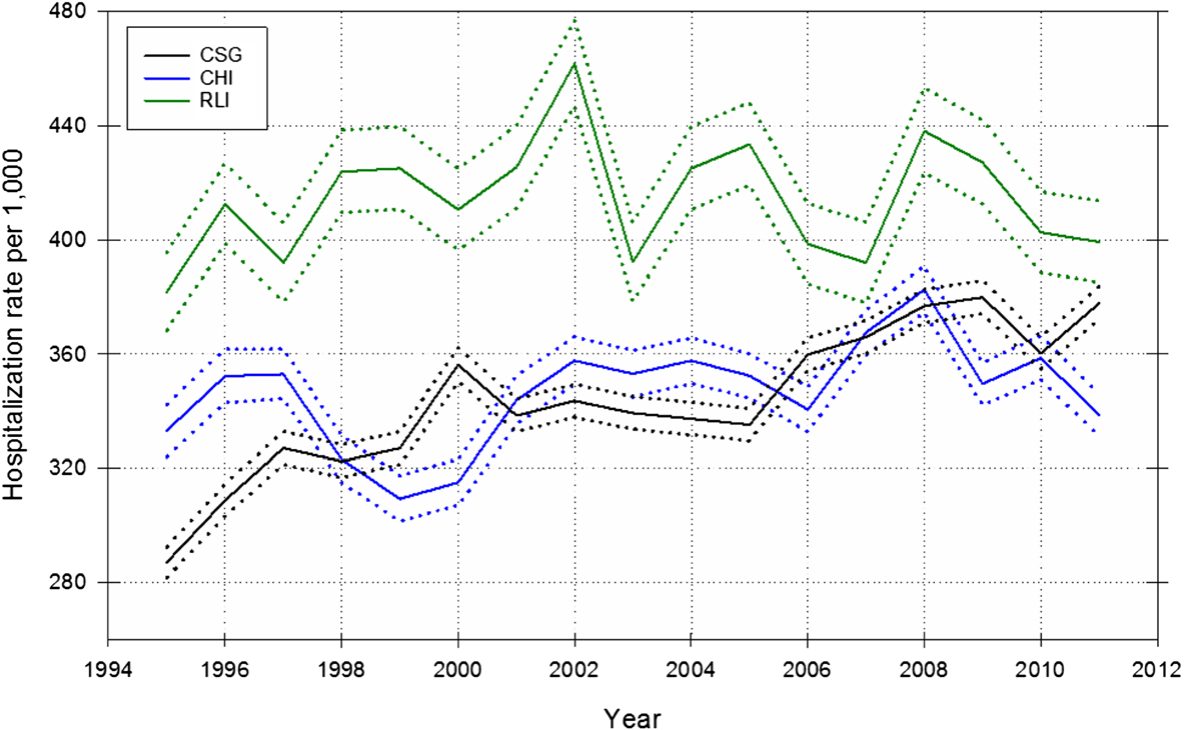
Age-standardized, all-cause hospitalization rates per 1000 with 95 % CI for the CSG, CHI, and RLI areas, 1995–2011. (Note: CSG = coal seam gas; CHI = coal high-impact; and RLI = rural low-impact)

**Fig. 3:**
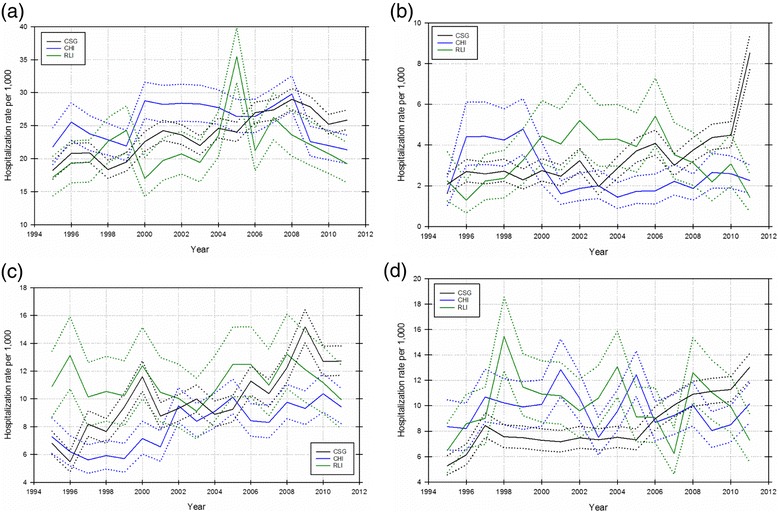
Age-standardized, cause-specific hospitalization rates per 1000 with 95 % CI for the CSG, CHI, and RLI areas, 1995–2011 for: **a** Chapter 2 (*‘Neoplasms’*); **b** Chapter 3 (*‘Diseases of the blood and blood-forming organs and certain disorders involving the immune mechanism’*); **c** Chapter 6 (*‘Diseases of the nervous system’*); and **d** Chapter 7 (*‘Diseases of the eye and adnexa’*). (Note: CSG = coal seam gas; CHI = coal high-impact; and RLI = rural low-impact)

There were 459 549 admissions to hospital from 1995 to 2011 across the three study areas, with 51.89 % from the CSG area, 35.83 % from the CHI area, and 12.28 % from the RLI area. Age-standardized hospitalization rates and 95 % CIs for *‘All-cause’* admissions are shown in Fig. 2. Age-standardized hospitalization rates by cause suggested increases over time in the CSG area relative to one or both study areas for health conditions described in ICD chapters relating to *‘Neoplasms’*, *‘Blood/immune’*, *‘Nervous system’*, and *‘Eye’* diseases (Fig. 3). Similar increases in the CSG area relative to the CHI and RLI areas were not evident for other ICD chapters.

Unadjusted models indicated there was a small, but significant increase over time in age-standardized, all-cause hospitalization rates in the CSG study area relative to *both* the CHI and RLI areas (see Table 2). This association was attenuated and not statistically significant following adjustment for all covariates.

**Table 2:** Rate ratios (RR) and 95 % CI for unadjusted and adjusted (age, sex, and additional covariates^a^) models for all-age hospitalizations in the coal seam gas, coal high-impact, and rural low-impact study areas over time (1995–2011)^b^

	CSG vs CHI	*p*-value	CSG vs RLI	*p*-value	CHI vs RLI	*p*-value
Unadjusted						
All-cause	1.01 (1.00–1.01)	0.009	1.01 (1.01–1.02)	0.0002	1.00 (1.00–1.01)	0.2195
Neoplasms	1.03 (1.01–1.04)	0.0016	1.01 (0.99–1.03)	0.3548	0.98 (0.97–1.00)	0.0448
Blood/immune	1.08 (1.05–1.12)	<0.0001	1.05 (1.01–1.09)	0.0096	0.97 (0.94–1.01)	0.1539
Nervous system	1.01 (1.00–1.03)	0.1198	1.04 (1.02–1.06)	0.0001	1.02 (1.00–1.04)	0.0204
Eye	1.04 (1.02–1.06)	0.0003	1.04 (1.02–1.06)	0.0002	1.00 (0.98–1.03)	0.7186
Adjusted (age and sex)						
All-cause	1.01 (1.00–1.01)	0.1178	1.01 (1.00–1.02)	0.0147	1.00 (1.00–1.01)	0.3655
Neoplasms	1.03 (1.00–1.05)	0.0167	1.00 (0.98–1.03)	0.7800	0.98 (0.95–1.00)	0.0521
Blood/immune	1.08 (1.04–1.12)	0.0001	1.05 (1.01–1.09)	0.0253	0.98 (0.93–1.02)	0.2701
Nervous system	1.01 (0.99–1.02)	0.5376	1.03 (1.01–1.05)	0.0015	1.02 (1.01–1.04)	0.013
Eye	1.04 (1.03–1.05)	<0.0001	1.04 (1.02–1.06)	<0.0001	1.00 (0.98–1.02)	0.8665
Adjusted (all covariates^a^)						
All-cause	1.02 (1.00–1.04)	0.1236	1.01 (0.99–1.04)	0.4044	0.99 (0.97–1.02)	0.6295
Neoplasms	1.01 (0.96–1.07)	0.6159	1.09 (1.02–1.16)	0.0091	1.07 (1.00–1.15)	0.0516
Blood/immune	1.08 (0.97–1.20)	0.1523	1.14 (1.02–1.27)	0.0025	1.05 (0.92–1.20)	0.4545
Nervous system	1.03 (0.99–1.08)	0.1715	0.99 (0.95–1.04)	0.7208	0.96 (0.91–1.01)	0.1531
Eye	1.03 (0.98–1.08)	0.2649	1.01 (0.95–1.06)	0.8028	0.98 (0.92–1.04)	0.4918

^a^Covariates in addition to age and sex included: proportion Indigenous; proportion Australian-born; proportion employed full-time; proportion white collar (managerial and administrative, professionals); weighted average of median household income; and weighted average of mean household size

^b^ *CSG* coal seam gas, *RLI* rural low-impact, *CHI* coal high-impact. RRs represent the slope change in a given area over time relative to the slope change in the comparison area. CSG is compared against the CHI reference group (Column 1) and the RLI reference group (Column 2). CHI is compared against the RLI reference group (Column 3)

For cause-specific hospitalization, adjusted models showed a 14 % (95 % CI: 1.02–1.27) increase in hospitalization rates for *‘Blood/immune’* diseases in the CSG area compared to the RLI area. Prior to adjusting for the covariates, the CSG area had rates that were 5 % higher than in the RLI area and 8 % higher than in the CHI area. Similarly, for hospitalization rates due to *‘Neoplasms’* in the adjusted model, there was a 9 % (95 % CI: 1.02-1.16) increase in hospitalization rates in the CSG area compared to the RLI area. Counts and 95 % CI for *‘Blood/immune’* diseases and *‘Neoplasms’*-related hospital admissions are shown in Additional file 1.

While *‘Nervous system’* disease-related admissions showed significant increases in the CSG area compared to the RLI area in the unadjusted models (RR: 1.04; 95 % CI: 1.02–1.06), this association decreased in the adjusted model and was no longer significant. Likewise, *‘Eye’* disease-related admissions increased significantly in the CSG area compared to the CHI (RR: 1.04; 95 % CI: 1.02–1.06) and RLI (RR: 1.04; 95 % CI: 1.02–1.06) areas in the unadjusted models; however, these associations were no longer significant after adjustment for all covariates.

## Discussion

The objective of this study was to assess potential health impacts of CSG development activities by examining increases in hospitalization rates for health conditions measured by ICD-10-AM chapter codes in three designated study areas (CSG, coal mining, rural) in Queensland over the period 1995–2011. CSG development activities only began to increase in 2001/2002 and steadily increased starting in 2005/2006 (see Fig. 1). To our knowledge, this is the first study of its kind in Australia.

In order to contextualize the results of the current analysis, available literature on UNGD impact evidence was reviewed to identify possible health conditions where increases in hospitalization rates due to CSG may be expected. Potential health outcomes included birth defects, cancer, cardiovascular outcomes, dermatological outcomes, injuries, neurological problems, psychosocial stress, respiratory disease, sexually transmitted infections, and vector-borne disease [2, 8, 13, 30–32]. These outcomes were matched with the appropriate ICD chapters where such outcomes would appear if a person were to be hospitalized. The identified ICD chapters are shown in Table 3, along with the ICD chapters where increases in hospitalization rates over time were observed in the CSG area relative to the CHI or RLI areas. Due to the scarcity of previously published data (generally, but also specifically within Australia), it was considered important to examine changes over time in hospitalization rates for all ICD chapters, not just those chapters matched from previous literature.

**Table 3:** Potential health outcomes associated with UNGD and corresponding ICD chapters from the literature, as well as the observed outcomes (unadjusted and adjusted) from this study for the coal seam gas (CSG) area

Potential outcome^a^	ICD chapter	Observed outcome (age-standardised)^b^
STIs	Infectious disease	
Vector-borne disease	Infectious disease	
Cancer	Neoplasms	X
Mental health	Mental disorders	
Neurological/nervous system	Nervous system	X^c^
Noise-related outcomes	Ear	
Cardiovascular outcomes	Circulatory	
Respiratory outcomes	Respiratory	
Dermatological outcomes	Skin	
Nephrotoxicity	Genitourinary	
Impaired fertility	Genitourinary	
Urological outcomes	Genitourinary	
Perinatal outcomes	Perinatal	
Birth defects	Congenital	
Injuries	Injuries	
n.d.	All-cause	X^c^
n.d.	Blood/immune	X
n.d.	Endocrine	
n.d.	Eye	X^c^
n.d.	Digestive	
n.d.	Musculoskeletal	

^a^Potential health outcomes identified in the literature. *n.d*. not determined in the literature

^b^Health outcomes identified in this chapter, where the CSG study area presented increases in hospitalization rates over time relative to the CHI and/or RLI areas

^c^This was significant in the unadjusted model; however, after adjusting for a number of covariates, this outcome was not significant

Other symptoms that were discussed in the literature were not included in Table 3 as they were either specific codes within chapters, or such outcomes would most likely fall within a group for which a person would not be admitted to hospital. This is true for many of the symptoms that have been reported (e.g., eye irritation, headaches, nosebleeds). Table 3 shows that a number of outcomes observed in the unadjusted and adjusted models in this study have not been previously mentioned in the literature as potential health outcomes related to UNGD (i.e., *‘Blood/immune’* and *‘Eye’* diseases).

Very few UNGD-related studies have examined hospitalization rates. One study examined all-age hospitalization rates for all-cause admissions across four counties with varying degrees of UNGD in the USA [33]. Garfield County, the county with the highest level of UNGD, was found to have the lowest or second lowest rate of all-cause hospitalizations. This finding is inconsistent with the present study, where no significant differences were found between areas, after adjustment of key demographic and socioeconomic characteristics. Another study examined hospital admissions alongside well number and density data [32]. While the study by Coons & Walker [33] used hospital admissions data over a 6.25-year period and the study by Jemielita [32] was over a 5-year period, our study was over a 17-year period.

The only overlap between health conditions identified in previous literature as being potentially associated with CSG, and for which increases in hospitalization rates were observed in the CSG area relative to another study area in the current analyses, was for neoplasms. Hospitalizations with a primary diagnosis code within this ICD chapter represent diagnosis of a neoplasm, where related codes (e.g., treatment) are within ICD chapters that were excluded from this study. However, this was not one of the strongest outcomes for all-age hospitalization rates because increases over time were noted in the CSG area compared only to the RLI area for adjusted models.

It is difficult to draw any conclusions with respect to possible changes in environmental exposures due to the fact that neoplasm trends typically reflect events 10–20 years prior to manifestation or are due to cumulative lifetime exposures [33]. Any short-term trends may not be reflective of changes in the health hazard impact potential of CSG development. Gas well development activity only began a steady increase in 2005/2006. Considering a very conservative lag period of 4 years [34], the *‘Neoplasms’* data presented here could only be reflective of changes after this period, with manifestation of disease after 2009/2010 (if the *‘Neoplasms’*-related diagnoses are related to any exposures associated with CSG development). Additionally, such changes can be an artefact of changes in screening practices [33].

While unrelated to UNGD, other health-related studies noted changes in hospitalization rates could be due to service changes in care for certain health outcomes (e.g., diabetes or pneumonia) [35, 36], changes in medical technology or laws [26], coding [35, 37, 38], or a combination of these factors [35]. Therefore, it could be hypothesized that the noted differences could be due to any of these changes, which have not been explored in this study. Such factors could also explain the increases in hospitalization rates that were noted in hospital admissions prior to the expansion of CSG development activities.

In these data, increases in hospitalization rates in the CSG area compared to the CHI *and* RLI areas were observed for *‘Blood/immune’* and *‘Eye’* diseases for unadjusted models. Adjusted models showed increases in hospitalization rates in the CSG area compared only to the RLI area (*‘Neoplasms’* and *‘Blood/immune’* diseases). All-age RR estimates were greatest for *‘Blood/immune’* disease-related admissions in the CSG area. Admissions within the *‘Blood/immune’* chapter include sub-chapters such as *‘aplastic and other anemias’*, *‘coagulation defects’*, *‘hemolytic anemias’*, *‘nutritional anemias’*, and *‘purpura and other hemorrhagic conditions’*. However, in absolute terms, admissions from this ICD chapter accounted for only 1.01, 0.52, and 0.79 % of each area’s total admissions for the CSG, CHI, and RLI areas, respectively (refer to Additional file 1).

The previously mentioned study by Coons & Walker [33] used Diagnostic-Related Groupings (DRG), whereas ICD chapters were used for this study. Therefore, the results are not directly comparable across all categories. For example, there is no equivalent *‘Blood/immune’* DRG that was used in the Coons & Walker study, only a *‘Red cell/clotting’* DRG category, which showed that rates decreased steadily over time in Garfield County. Likewise, the study by Jemielita et al. [32] did not find any significant associations for the *‘Hematology’* category.

These findings are dissimilar to those presented here, even after adjusting for covariates. Diseases from this ICD chapter (e.g., anemia and other blood disorders) have been discussed in the UNGD literature in relation to worker health and exposure to benzene, toluene, ethylbenzene, and xylene (BTEX) [2]; however, such discussion is lacking in terms of community health. Generally, long-term exposure to benzene most often affects the blood, and such exposure can also affect the immune system [39], for which such outcomes are found in the *‘Blood/immune’* chapter. The most common route of exposure to BTEX is through inhalation, typically through air contaminated by motor vehicle emissions and industrial use, as well as cigarette smoke [40]. While BTEX compounds are naturally occurring and can be found in some water sources, the Queensland Government now has laws in place that ban the use of such compounds in hydraulic fracturing fluids [41].

Sub-chapters within the *‘Eye’* ICD chapter include *‘disorders of the eyelid, lacrimal system and orbit’*, *‘disorders of conjunctiva’*, *‘disorders of lens’*, *‘glaucoma’*, *‘disorders of vitreous body and globe’*, and *‘visual disturbances and blindness’*, amongst others. In relation to the Coons & Walker [33] study that used the DRG category for diseases of the eye, hospitalization rates were lowest in Garfield County [33]. Additionally, there were no significant findings within the *‘Ophthalmology’* category used by Jemielita et al. [32] Both of these findings are in contrast with the results from the unadjusted models for the *‘Eye’* disease-related hospitalization rates presented here; however, the results are similar (i.e., no significant findings) after adjusting for covariates.

Numerous studies have raised the issue of eye-related symptoms, such as burning, irritation or itching, associated with UNGD [3, 42–45]; however, these studies have discussed outcomes in terms of self-reported symptoms rather than eye-related diseases for which a person would be admitted to hospital. In discussing UNGD operations, Brown et al. [30] noted that short-term exposure to volatile organic compounds can irritate the eyes, and exposure to diesel emissions can also cause eye irritation. The data presented here would capture the most severe cases rather than residents reporting the symptoms that have typically been discussed in the literature.

In terms of chemicals affecting these systems, Colborn et al. [46] assessed chemicals used in UNGD operations and found that more than 75 % of the chemicals assessed can affect sensory organs such as the eyes. Likewise, 40 % of chemicals can affect the immune system and 46 % can have possible health effects on the cardiovascular system and blood [46]. However, it must be noted that this analysis focused on chemicals used in UNGD operations in the United States, which also includes shale gas. Hence, it was unclear whether chemicals used specifically in CSG operations were included in the analyses.

This study has several limitations. Firstly, the study is limited by the ecologic approach used and the associated possibility of ecologic fallacy. While individual, episode-based hospital admissions data were provided, the data were grouped according to the three geographic areas (classified by varying levels of environmental impact), which served as a proxy for unmeasured exposures. Grouping of data in this manner limited the analyses, and represented the smallest aggregations of geographic areas that were allowable by Queensland Health, due to privacy and confidentiality concerns. The unadjusted models showed there were increases in specific hospital admissions in the CSG area relative to the other two study areas; however, these increases were modest and RR estimates were generally small and confidence intervals generally narrow. After adjustment for all covariates, increases in admissions in the CSG area were significantly higher compared only to the RLI area for certain outcomes. Due to small sample sizes for some of the ICD chapters within given study areas, these results should be interpreted cautiously.

The hospital admissions database represents the highest level of morbidity data available, meaning that any data below this (i.e., General Practitioner (GP) or Emergency Department visits) were not captured. There is also lack of data on the percentage of people who do not seek health care in the three study areas, so true rates of health impact are likely to be underestimated. In addition, hospitalization data are episode-based and not person-based, hence, repeat admissions were included in this dataset. Therefore, a resident could have been admitted for the same primary diagnosis on more than one occasion within the same year.

Additionally, it is possible that residents moved from one area to another, moved out of the area entirely, or died, which could result in measurement errors. While we obtained hospital admissions data only on residents of the three study areas to exclude admissions of non-resident workers, it is possible that non-resident workers were included in population enumeration for these areas, depending on a worker’s interpretation of ‘usual residence’ [25, 47]. Considering this, the rates could be underestimated for residents of the three study areas. While empirical data on the impact of fly-in, fly-out workers on health services is lacking [48], a recent report found that non-resident workers did have a significant impact, with up to 30 % of health service presentations coming from non-residents [49]. This may not be applicable to all mining communities; therefore it was suggested community-specific analyses be conducted by collecting and including the home address postcode for each patient, along with diagnoses [48]. These are the methods that were used in our analyses, although data were collected by broader home address groupings for each patient due to limitations previously addressed.

Certain indicators have been linked to poorer health such as income, household size and overcrowding, and education attainment [50, 51]. Indigenous Australians also have poorer health outcomes, with one of the highest levels of health inequality compared to Indigenous groups [52] and a disproportionate level of chronic disease compared to non-Indigenous Australians [53]. While our adjusted model controlled for factors that were available across the entire time period (e.g., proportion employed full-time was available across all Census years, but a uniform measure of education attainment was not), these were ecological adjustments for the demographic and socioeconomic factors given and were necessarily based on the geographic unit of analysis provided in the hospital admissions data.

The analyses presented in this paper do not allow for conclusions that CSG is a cause of any of the increased hospitalization rates reported. The present study was a descriptive-analytic study employing ecologic units of analysis using routinely available health indicator data. This study provides a preliminary assessment of hospitalization rates and serves to generate hypotheses for future research. As such, the results presented here suggest areas that should be explored further with more sophisticated study designs, and using higher resolution data (e.g., Emergency Department presentations, presentations to GPs) than what could be obtained for this study. Additionally, CSG development in Australia is a contentious issue [1, 17, 54], and much of the data that are collected are predominantly used for legislative compliance purposes rather than monitoring and research purposes [17]. Calls have been made for more environmental data, as well as health data, that are publicly available in a common repository [17].

Further examination of these hospitalization data to determine trends over time in age-specific and gender-specific rates of the health conditions potentially related to CSG is recommended. It would be useful to include data on the working population of an area, where study time periods allow for inclusion of such data, to better understand populations with a high proportion of fly-in, fly-out workers. In addition, analyses on specific diseases identified in previous literature for which potential health outcomes may arise should be examined. Further research using robust methodology is required to investigate the potential causal association between CSG and the potential adverse health outcomes presented here.

## Conclusions

The findings from this preliminary study suggest an increase in hospitalization rates over time for some broad primary diagnoses by ICD chapter after accounting for key demographic and socioeconomic factors for the CSG study area compared to the CHI and/or RLI study areas. Strongest observed associations were found for *‘Blood/immune’* diseases. Analyzing age- and gender-specific rates, as well as specific sub-chapters within the main ICD chapter headings, are important next steps. While this study makes no attempt to attribute causality, the results of this study suggest areas that should be explored further.

## Additional file

Additional file 1:
**Number of admissions and 95 % confidence intervals for the CSG, RLI, and CHI study areas for** ***‘Blood/immune’*** **disease and** ***‘Neoplasms’*** **-related admissions** ^**a**^ **.** (DOCX 18 kb)

## Abbreviations

ABS: Australian Bureau of Statistics
CHI: Coal high-impact
CI: Confidence interval
CSG: Coal seam gas
DRG: Diagnostic-Related Groupings
ERP: Estimated resident population
GP: General Practitioner
HIA: Health Impact Assessment
ICD: International Classification of Diseases
QHAPDC: Queensland Hospital Admitted Patient Data Collection
RLI: Rural low-impact
RR: Rate ratio
SLA: Statistical Local Area
UNGD: Unconventional natural gas development
