# Supplementary material for: All-age hospitalization rates in coal seam gas areas in Queensland, Australia, 1995–2011
Source: BMC Public Health. 2016 Feb 6;16:125. doi: 10.1186/s12889-016-2787-5 (PMC4744625; doi:10.1186/s12889-016-2787-5)
Supplement: Additional file 1: — Number of admissions and 95 % confidence intervals for the CSG, RLI, and CHI study areas for ‘Blood/immune’ disease and ‘Neoplasms’ -related admissions a . (DOCX 18 kb) [file 12889_2016_2787_MOESM1_ESM.docx]

| Additional file 1. Number of admissions and 95% confidence intervals for the CSG, RLI, and CHI study areas for *‘Blood/immune’* disease and *‘Neoplasms’*-related admissions.^a^ | | | | | | | |
| --- | --- | --- | --- | --- | --- | --- | --- |
|  |  | **CSG** | | **RLI** | | **CHI** | |
|  |  | n | 95% CI | n | 95% CI | n | 95% CI |
| *‘Blood/immune’* | 1995 | 76 | 60-95 | 16 | 9-26 | 39 | 28-53 |
|  | 1996 | 97 | 79-118 | 12 | 6-21 | 56 | 42-73 |
|  | 1997 | 95 | 77-116 | 18 | 11-28 | 52 | 39-68 |
|  | 1998 | 101 | 82-123 | 18 | 11-28 | 70 | 55-88 |
|  | 1999 | 88 | 71-108 | 25 | 16-37 | 73 | 57-92 |
|  | 2000 | 102 | 83-124 | 35 | 24-49 | 49 | 36-65 |
|  | 2001 | 96 | 78-117 | 31 | 21-44 | 45 | 33-60 |
|  | 2002 | 133 | 111-158 | 42 | 30-57 | 48 | 35-64 |
|  | 2003 | 82 | 65-102 | 34 | 24-48 | 46 | 34-61 |
|  | 2004 | 119 | 99-142 | 34 | 24-48 | 30 | 20-43 |
|  | 2005 | 158 | 134-185 | 31 | 21-44 | 37 | 26-51 |
|  | 2006 | 175 | 150-203 | 44 | 32-59 | 37 | 26-51 |
|  | 2007 | 129 | 108-153 | 28 | 19-40 | 49 | 36-65 |
|  | 2008 | 166 | 142-193 | 25 | 16-37 | 48 | 35-64 |
|  | 2009 | 193 | 167-222 | 17 | 10-27 | 57 | 43-74 |
|  | 2010 | 200 | 173-230 | 25 | 16-37 | 60 | 46-77 |
|  | 2011 | 403 | 365-444 | 11 | 5-20 | 59 | 45-76 |
| *‘Neoplasms’* | 1995 | 685 | 635.59-738.25 | 134 | 112.27-158.71 | 371 | 334.21-410.75 |
|  | 1996 | 778 | 725.22-834.62 | 151 | 127.88-177.10 | 499 | 456.15-544.71 |
|  | 1997 | 787 | 733.90-843.94 | 151 | 127.88-177.10 | 485 | 442.83-530.09 |
|  | 1998 | 701 | 650.99-754.85 | 176 | 150.96-204.01 | 513 | 469.56-559.41 |
|  | 1999 | 756 | 704.00-811.85 | 191 | 164.87-220.10 | 491 | 448.52-536.47 |
|  | 2000 | 876 | 819.88-935.96 | 132 | 110.45-156.54 | 587 | 540.49-636.45 |
|  | 2001 | 967 | 907.94-1029.90 | 152 | 128.80-178.17 | 561 | 515.59-609.40 |
|  | 2002 | 967 | 907.94-1029.90 | 164 | 139.86-191.12 | 548 | 503.08-595.87 |
|  | 2003 | 912 | 854.70-973.14 | 155 | 131.56-181.41 | 551 | 506.00-598.94 |
|  | 2004 | 1046 | 984.50-1111.34 | 187 | 161.16-215.80 | 567 | 521.24-615.68 |
|  | 2005 | 1045 | 983.53-1110.31 | 291 | 258.52-326.44 | 554 | 508.83-602.04 |
|  | 2006 | 1162 | 1097.08-1230.76 | 175 | 150.03-202.94 | 576 | 529.95-625.05 |
|  | 2007 | 1200 | 1134.00-1269.84 | 217 | 189.09-247.87 | 617 | 570.20-667.64 |
|  | 2008 | 1296 | 1227.34-1368.51 | 192 | 165.80-221.17 | 653 | 604.80-705.04 |
|  | 2009 | 1251 | 1183.57-1322.27 | 179 | 153.74-207.23 | 526 | 481.99-572.88 |
|  | 2010 | 1141 | 1076.69-1209.15 | 173 | 148.18-200.79 | 508 | 464.76-554.23 |
|  | 2011 | 1205 | 1138.86-1274.98 | 163 | 138.93-190.04 | 508 | 464.76-554.23 |
| ^a^ Note: CSG = coal seam gas; RLI = rural low-impact; and CHI = coal high-impact. | | | | | | | |
